# Glucagon and other proglucagon-derived peptides in the pathogenesis of obesity

**DOI:** 10.3389/fnut.2022.964406

**Published:** 2022-08-04

**Authors:** Jens Juul Holst

**Affiliations:** The NovoNordisk Foundation Center for Basic Metabolic Research, Department of Biomedical Sciences, Faculty of Health Sciences, University of Copenhagen, Copenhagen, Denmark

**Keywords:** glucagon-like peptide-1 (GLP-1), peptide YY, oxyntomodulin, glicentin, gut hormones, proglucagon

## Abstract

Because of differential processing of the hormone precursor, proglucagon, numerous peptide products are released from the pancreatic alpha cells and the intestinal L-cells in which the (pro)glucagon gene is expressed. Of particular interest in relation to obesity are glucagon from the pancreas and oxyntomodulin and GLP-1 from the gut, all of which inhibit food intake, but the other products are also briefly discussed, because knowledge about these is required for selection and evaluation of the methods for measurement of the hormones. The distal intestinal L-cells also secrete the appetite-inhibiting hormone PYY. Characteristics of the secretion of the pancreatic and intestinal products are described, and causes of the hypersecretion of glucagon in obesity and type 2 diabetes are discussed. In contrast, the secretion of the products of the L-cells is generally impaired in obesity, raising questions about their role in the development of obesity. It is concluded that the impairment probably is secondary to obesity, but the lower plasma levels may contribute to the development.

## Introduction

The hormones derived from proglucagon are currently receiving considerable interest in relation to obesity research since at least three of these, glucagon itself, glucagon-like peptide-1 (GLP-1) and oxyntomodulin, have pronounced effects on appetite and food intake and form the basis for drugs for the treatment of obesity (1). Indeed, some of the GLP-1 receptor agonists, are the most efficacious (approved) weight-lowering agents known. It is therefore of interest to study the secretion from their sites of origin in health and obesity to see whether abnormalities of secretion may be involved in the development of obesity. An analysis of our knowledge regarding the secretion and possible actions of the *endogenous* proglucagon products in obesity is the purpose of this brief overview.

### Proglucagon

**Figure 1:**
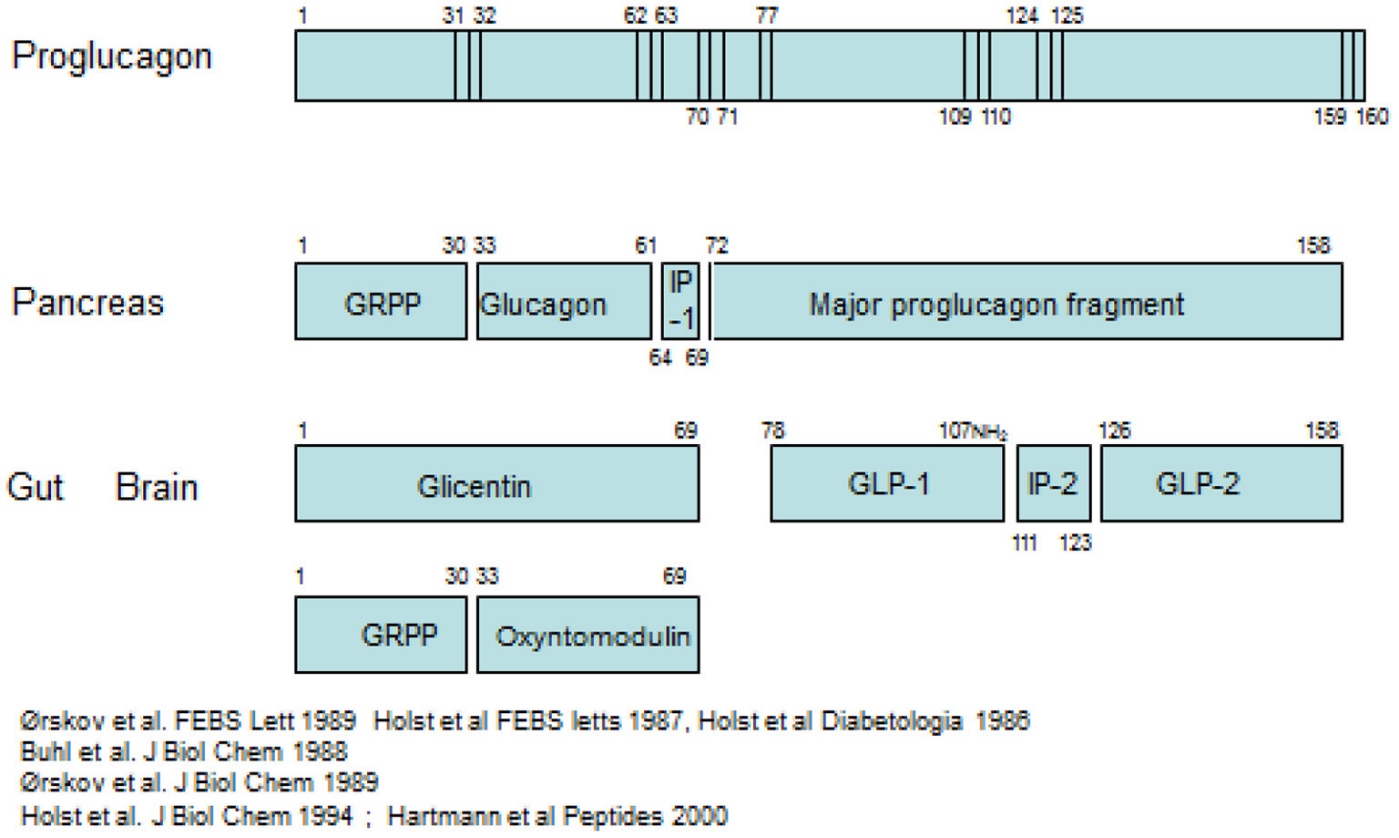
Processing of proglucagon in the pancreas and the gut.

The pancreatic hormone glucagon, is produced in the alpha cells of the pancreas by cleavage of the precursor, proglucagon, catalyzed by the prohormone convertase PC2 as indicated schematically in Figure 1 (2, 3).

Proglucagon is a peptide of 160 amino acids of which glucagon occupies positions 33–61. Importantly, when the alpha cell is stimulated to secrete glucagon, all the remaining products of proglucagon are also thought to be released. The glucagon gene is also expressed in L-cells (4), endocrine cells of the intestinal epithelium, and it is from these cells that the hormone GLP-1 is cleaved from proglucagon, mediated by another prohormone convertase, PC1/3. GLP-1 occupies positions 78–108 in proglucagon (5). As in the pancreas, the remaining parts of the proglucagon molecule are also released in parallel with GLP-1 (6). Identification of the products of proglucagon processing requires accurate chemical analysis of the various products, since the processing cannot be predicted: several prohormone convertases may be involved; one cannot rely on their predicted substrate specificity; and there may be also some expression of PC2 in the gut and of PC1/3 in the alpha cells. As a general rule, cleavage occurs where a pair of basic amino acid residues (lysine, arginine) is found, but a look at the sequence of proglucagon reveals that not all of these sites are cleaved and that PG is also cleaved at other sites, for instance at a single basic residue. Examples are the basic amino acids at position 17–18 in glucagon, which are not cleaved, and the monobasic residue no 77 in PG which *is* cleaved, apparently by PC1/3 (7).

### Pancreatic processing of proglucagon

The processing pattern and subsequent release of the products from the pancreas has been demonstrated directly with assays for the N-terminal fragment, GRPP (glicentin-related pancreatic polypeptide) and assays for glucagon in effluent from isolated perfused preparations of the pancreas where this can be conveniently studies without interference from metabolites formed in the body (6, 8). A small amount of PG 1–61 may also be secreted (9). Such studies also indicate that the C-terminal part of the precursor, the so-called Major Proglucagon Fragment (10), corresponding to residues no. 72–160 in PG, is also released without further cleavage (6). Very little is known about MPGF; thus, it is not known whether it is broken down in the circulation, how it is metabolized or whether it may have biological activity. In principle, it should be measurable by side-viewing assays for GLP-1 and GLP-2, but it may not survive pre-analytical procedures employed for these assays (11). A small amount of MPGF appears to be cleaved already in the alpha cells resulting in formation and secretion of proglucagon 72–108 (GLP-1 1–37) and GLP-2 (PG 126–158) (2). Even smaller amounts of GLP-1 7–36 amide may also be secreted and are particularly observed in pancreases with alpha cell hyperplasia (12). Of these peptides, only glucagon itself is supposed to influence appetite and food intake (13), whereas neither PG 1–61, PG 72–108, or GLP-2 are thought to have significant effects. In accordance with the processing profiles described, relevant estimations of the pancreatic potential to release appetite suppressors can be obtained by measuring plasma concentrations of glucagon. This can be done reliably by sandwich ELISAs incorporating terminal wrapping antibodies (14), whereas other antibodies may crossreact with the intestinal PGDPs, glicentin and oxyntomodulin, a fact which has generated considerable confusion in the past. Particularly in rodents, pancreatic release of PG 72–107 amide (i.e., N-terminally extended GLP-1, also called GLP-1 72–78 amide) has caused confusion because it is co-released with glucagon and may be picked up by insufficiently specific GLP-1 assays. The stress of blood sampling or drug administration in conscious rodents [which results in glucagon secretion (15)] may cause a release of this peptide. It should be mentioned that an assay for MPFG has recently been developed by the company Ansh Labs, but the experience with this assay is so far very limited.

### Intestinal processing of proglucagon; other L-cell products

The processing in the gut differs markedly and results in (Figure 1) formation of PG 1–69 from the N-terminal part, aka glicentin, which may be broken down to GRPP (as in the pancreas) and oxyntomodulin (PG 33–69) (8, 16). From the C-terminal part, the processed and secreted products predominantly comprise PG 77–107 amide (GLP-1) and PG 126–158 (GLP-2), although intermediate forms may also be found (6). The so-called intervening peptide 2 (PG 111–123) is also formed and secreted, but nothing is known about this interesting peptide, which is also C-terminally amidated (17). Again, GRPP is not known to have biological activity (and also shows much more sequence variation between species than the other forms—GLP-1, for instance, is invariant in mammals). Also glicentin (18) may be inactive, whereas all of oxyntomodulin, GLP-1, and GLP-2 are biologically active and have been reported to influence appetite and food intake (3, 19). It should be mentioned here that a large part of especially the distal L-cells also express and secrete peptide YY 1–36, which after cleavage by DPP-4 in the circulation to the 3–36 form is a potent appetite suppressor (20). The L-cell thus may liberate no <3 potent inhibitors of food intake.

### Hormonal products of the intestinal L-cells

Obviously, the potential of the L-cell for regulation of appetite and food intake should be evaluated on the basis of estimates of the secretion of all of the three active peptides, but this is rarely done. Under normal conditions, the release of oxyntomodulin, which always represents a fraction of glicentin is presumably limited, since it acts on the glucagon and the GLP-1 receptors, but at only 1/100 of their potency (21). Regarding GLP-1 and PYY, both peptides are rapidly degraded by the ubiquitous enzyme DPP-4, which for GLP-1 means formation of the metabolite GLP-1 9–36 amide, which is a weak antagonist at the GLP-1 receptor (22). It has been calculated that only about 10% of the GLP-1 originally released from the L-cells reaches the arterial circulation in the intact form (23, 24). For PYY, on the other hand, this conversion from PYY 1–36 to PYY 3–36 means *activation* because (only) the metabolite can interact with the Y2 receptor, which transmits satiety in the hypothalamus (25). Probably around half of the circulating PYY is metabolized by DPP-4, but additional enzymatic systems quickly degrade PYY also from the C-terminus, generating PYY 3–35 or 3–34, both of which are inactive (26, 27). Potential PYY action on Y2 receptors, therefore, can only be expected for a very short period after its release. However, it is likely that both peptides interact with sensory afferent nerve fibers of the parasympathetic innervation already in the gut and that their physiological impact on the brain stem and hypothalamus is mediated by these fibers (3, 28). So how is it possible to estimate the impact of L-cells secretion? For GLP-1, the recommended way is to measure “total GLP-1” comprising the sum of both intact GLP-1 and the metabolite, GLP-1 9–36 amide, assuming that the GLP-1 metabolite had the possibility of interacting with the nerve fibers in the gut wall before it was degraded by DPP-4 (29). Because of the extensive and rapid degradation, it is of little use to measure “intact GLP-1” (although in experimental animals it may be possible to pretreat the animals with inhibitors of DPP-4 and preferably also neprilysin and then measure GLP-1 with an assay for intact GLP-1). For PYY, it is probably best to measure plasma levels of PYY3–36. However, available PYY assays do not distinguish between intact PYY 3–36 and the C-terminally truncated forms (27). Presumably, with the newer mass spectrometry based methods, it may be possible to measure intact PYY (3–36), which would be highly desirable.

## Secretion in obesity

### Glucagon

Glucagon secretion (30, 31) must be evaluated based on three characteristics: (1) the secretion in the fasting state; (2) estimation of stimulated secretion; and (3) evaluation of suppressibility of secretion.

In the fasting state, there is an active secretion of glucagon. It is important because of its role in maintaining the fasting levels of glucose in plasma. In this respect, it is very different from the gut hormones, which most likely mainly have impact when they are secreted in response to meal ingestion. There are several sets of stimuli for glucagon secretion, the most important are: responses to hypoglycemia, to protein ingestion/amino acids and to sympathetic stimulation. Glucagon secretion capacity may be evaluated from amino acid infusion tests; most often arginine is injected intravenously in various doses, typically 0.5 g, but this is usually done in relation to tests of beta cell function and not in a systematic way for glucagon evaluation. *Suppressibility* of glucagon secretion is considered an important parameter for glucose tolerance and is most often studied during a 75 oral glucose tolerance test. People with normal glucose tolerance suppress their plasma glucagon concentration to very low levels (a few pmol/L) within an hour, and this is one of the mechanisms behind the decrease in hepatic glucose production, which is required for normal glucose tolerance. People with impaired glucose tolerance/type 2 diabetes, typically have elevated fasting levels of glucagon and may show increasing levels of glucagon during the 1st h of the OGTT and the suppression is typically delayed, but the same low levels are normally reached after 2–3 h. Although not fully established, recent research has thrown some light on these patterns and has directed the focus toward the so-called liver-alpha cell axis (32), where glucagon acts on the liver to regulate amino acid metabolism and thereby their plasma levels, while the amino acids in turn stimulate glucagon secretion in a close feed-back cycle. The function of the axis very clearly shows up in conditions with very high rates of glucagon secretion (glucagonoma syndrome), where amino acids reach dramatically low plasma levels with deleterious consequences for cell turnover, for instance in the skin where cells die and give rise to the so-called necrolytic migratory erythema. In people with inactivating mutations of glucagon receptor, amino acid levels rise to high levels and are responsible for extreme hyperglucagonemia and alpha cell hyperplasia (33). It now appears that the high fasting levels of glucagon in patients with T2DM are more related the occurrence of NAFL and steatosis of the liver, which causes resistance to the actions of glucagon with resulting hyperaminoacidemia. This, in turn, causes increased glucagon secretion and hyperglucagonemia both in the fasted state and postprandially (it should be noted that the effects of a mixed meal depends on its content of protein (stimulating glucagon secretion) vs. carbohydrates (inhibiting secretion). In agreement with this view, hyperglucagonemia is also observed in non-diabetic obesity, provided this is associated with increased liver fat (34). The liver fat also causes insulin resistance, and the combined impairment of the actions of glucagon on amino acid metabolism in the liver, while its effect on glucose production are preserved, and the insulin resistance, probably are responsible for the fasting hyperglycemia in T2DM (32).

### L-cell secretion in obesity

**Figure 2:**
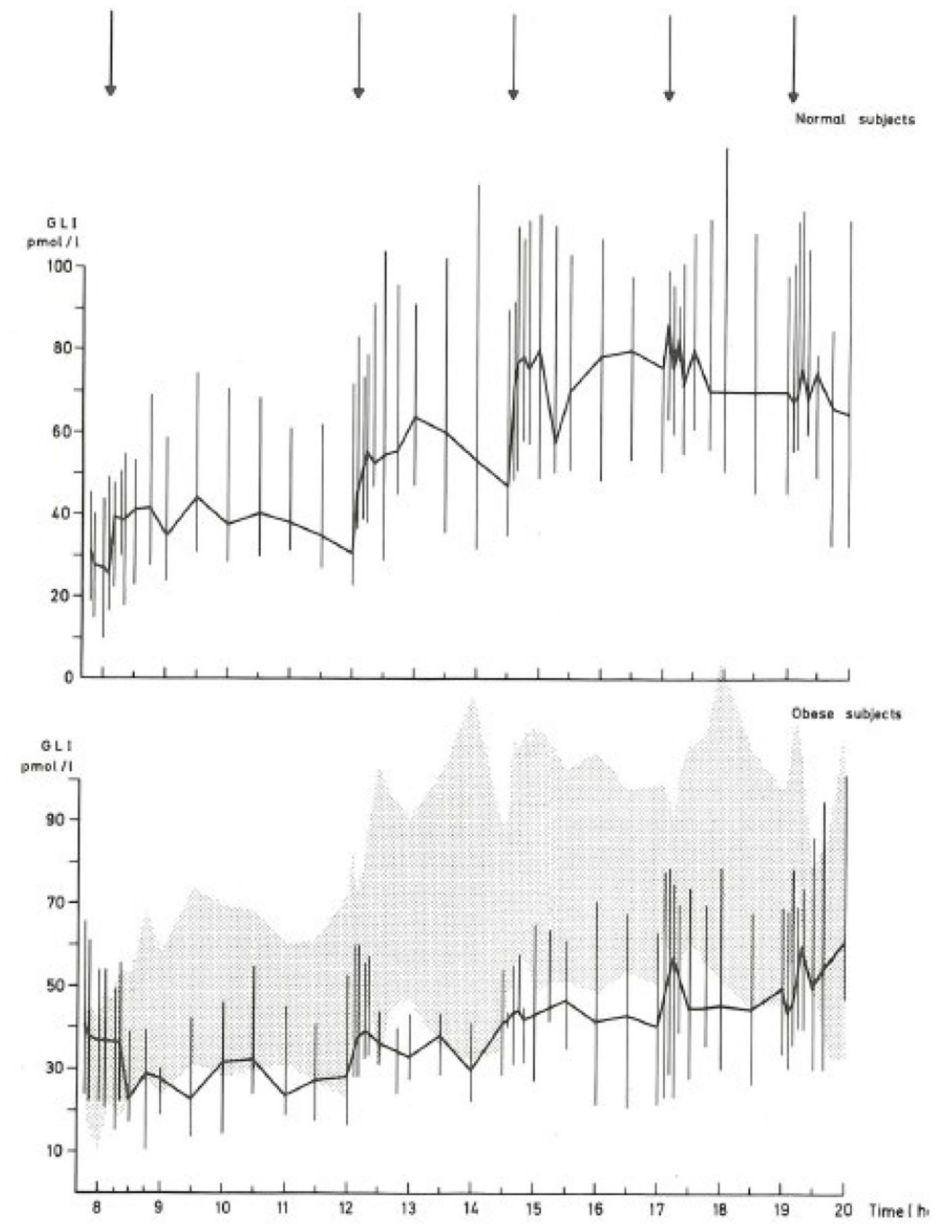
Diurnal profile of enteroglucagon (glicentin + oxyntomodulin) in plasma from lean and morbidly obese subjects throughout the day (38). Full lines: median values; vertical bars: total range; shaded area: total range (= 95% confidence intervals) of healthy subjects. Arrows: meals.

**Figure 3:**
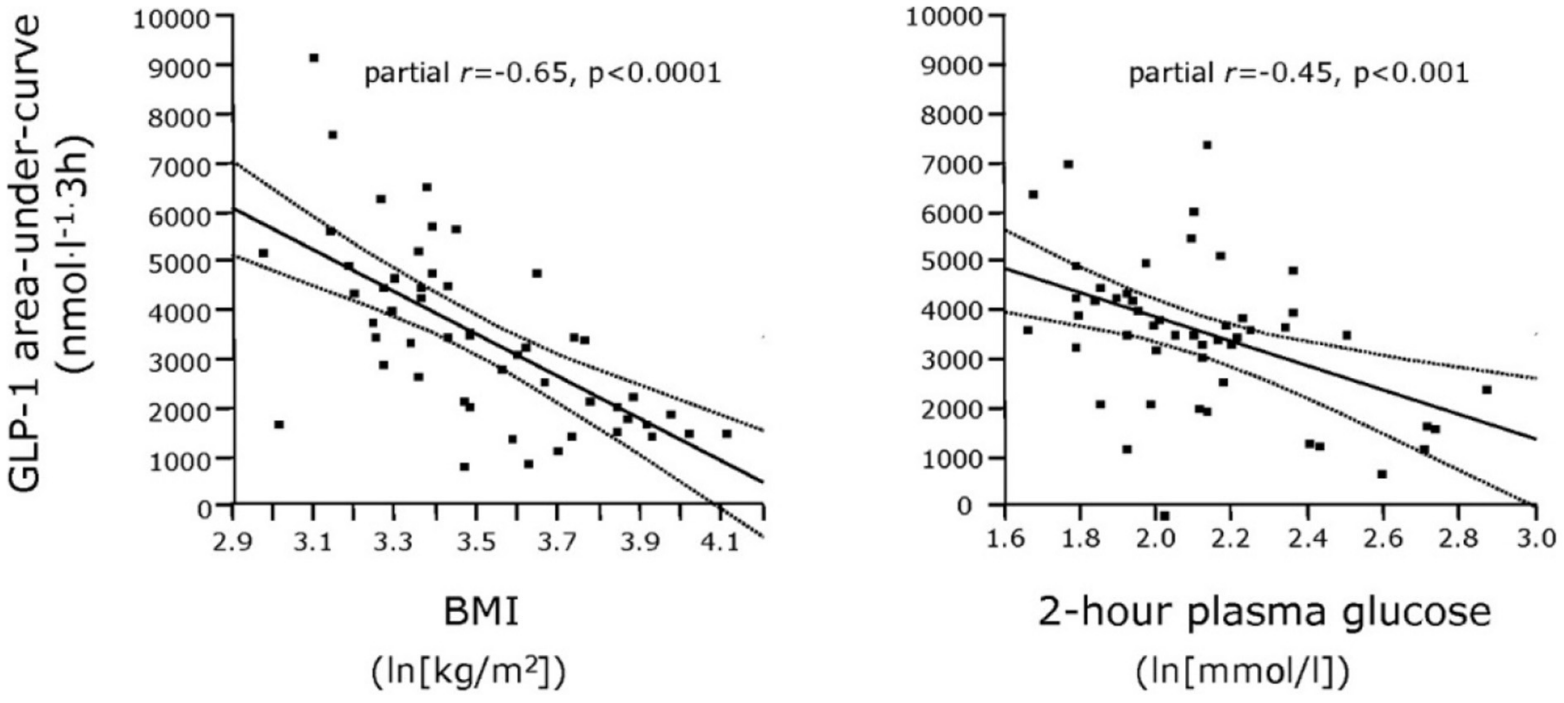
Relationship between plasma GLP-1 responses (Areas Under Curve) and BMI as well as 2-h plasma glucose concentrations in patients with varying degrees of obesity and T2DM. From Muscelli et al. (39).

The literature concerning L-cell secretion is quite extensive since it dates back to the early measurements of gut glucagon-like immunoreactivity, now known to represent glicentin and oxyntomodulin. Of the gut-derived products of proglucagon, glicentin probably has the longest half-life, and may therefore provide the best reflection of the L-cell activity. Oxyntomodulin levels are, as mentioned, low (21) and difficult to measure accurately since this requires sandwich ELISAs with terminal wrapping antibodies reacting with the N-terminus of the glucagon part and the C-terminus of the glicentin tail. A prototype of such an assay has been developed by Mercodia, and measurements with this assay are in agreement with results obtained with RIAs involving cross-reacting glucagon antibodies and chromatography (35). GLP-2 has not been studied a lot, mostly because very few reliable assay are available. In general, and in agreement with the simultaneous exocytosis of GLP-1 and GLP-2, and because the elimination rates of GLP-2 and the GLP-1 metabolite (GLP-1 9–36 amide) are similar, GLP-2 values follow (total) GLP-1 levels (36). Since the discovery of the therapeutic potential of GLP-1 in diabetes and obesity, numerous studies have been performed in people with obesity and/or T2DM (37). Early indications of severely lowered diurnal secretion in obesity were obtained with assay for enteroglucagon (crossreacting glucagon antibodies) in people with morbid obesity (38) (see Figure 2), and early measurements of total GLP-1 in obese individuals supported this conclusion (see Figure 3) (39, 40).

Much later, in a large cohort study of 1,462 individuals with normal glucose tolerance, prediabetes, or screen-detected diabetes, obese and overweight subjects had 20 and 15% reduced GLP-1 responses to an OGTT after adjustment for age, sex, and glucose status (41). In a comprehensive twin study from Finland, it was possible to study both mono- and dizygotic twins concordant and discordant for obesity to determine the heritability of GLP-1 responses to OGTT and the influence of acquired obesity on GLP-1 responses during OGTT or meal tests (42). While the GLP-1 response to the OGTT was clearly heritable, an acquired unhealthy pattern of obesity, characterized by liver fat accumulation and insulin resistance, was closely related to impaired GLP-1 responses in young adults. The influence of obese individuals with type 2DM, was evaluated in an early study from 2001, which showed a preserved intact early response, but an impaired subsequent response in the patients compared to a weight- matched control group and a group of people with impaired glucose tolerance, who had intermediate responses (43). Because impaired GLP-1 responses might be partly responsible for the impaired incretin effect in people with T2DM, this was an important observation, but other most often smaller studies did not confirm the impaired secretion and meta-analyses have not been able to conclude that impaired secretion is a general finding (44, 45). In addition, it turned out that the impaired incretin effect in T2DM is mainly due to severely impaired insulinotropic effects, not only of GLP-1, but in particular of the other incretin hormone GIP, which in some T2DM patients has completely lost insulin-tropism (46, 47). The lower secretion in obese individuals has been confirmed in many studies, and combined with the finding that also PYY levels are reduced in obesity (48), supports the notion of an impairment of L-cell performance in obesity. It has been demonstrated that weight losses are associated with improved secretory responses (40).

It is of course important to explore whether the lower secretion of PYY and GLP-1 in obesity is a consequence or a cause of the condition. One fundamental problem is how the secretory response should be studied. It is usually done by comparing results of either an OGTT or mixed meal tests in lean and obese individuals. This raises the question whether the heavy and the lean individuals should have the same meal. Both PYY and GLP-1 responses depend on the meal size (25, 49) and if the meal size is related to body weight, the heavier individuals would have a much larger meal. With identical meals, lean subjects get a proportionally larger meal, which could explain the lower responses in the heavy individuals. One could also argue that the meal size should be calculated to correspond to the basal energy expenditure to reflect the nutritional need of the individual. Unfortunately, studies designed to elucidate these problems have not been done. Recently a rodent study showed that the intrinsic capacity to secrete gut hormones (from isolated gut segments) was uninfluenced by presence/absence of obesity suggesting that the lower secretion is not due to differences in the secretory characteristics of the L-cells (Jepsen In Press Frontiers).

Unfortunately, there are no longitudinal studies to provide information about the time when, in the development of obesity, the impaired secretion of PYY and GLP-1 sets in. As will be understood, it is difficult to assign a causal role for the development of obesity to the decreased secretion of GLP-1 and PYY. Currently, the evidence would be compatible with an impairment that develops with the condition. However, the physiological role of PYY and GLP-1 in appetite and food intake regulation seems well-established by now (20, 37), and the decreased secretion in obesity would therefore seem to contribute negatively to the condition. On this background, it seems reasonable to treat obesity with these hormones. Indeed, co-administrations of PYY and GLP-1 and most recently also combined with oxyntomodulin have been demonstrated to profoundly influence food intake (50, 51) and body weight (52), and the long acting GLP-1 agonists now result in two digit weight losses (53). A very powerful GIP/GLP-1 co-agonist, tirzepatide is currently under clinical development; this compound results in large weight losses in people with diabetes (54) and losses exceeding 20% in people with obesity (Lilly, press release May 2022). This raises the question about the role of GIP in obesity. However, in humans, GIP is normally considered to promote nutrient uptake and deposition and neither has an inhibitory effects on appetite or food intake when given alone, nor when combined with GLP-1 (55). Thus, one current view is that tirzepatide mainly performs as a potent GLP-1 analog. Indeed, it has no effect on body weight in mice with genetic deletion of the GLP-1 receptor in the CNS (56).

## Conclusion

There is general agreement that the secretion of intestinal proglucagon derived appetite inhibitory hormones, GLP-1, PYY, and oxyntomodulin is impaired in obesity. This does not seem to be due to an intrinsic L-cell defect, and most likely the impairment develops as a consequence of obesity. Thus, the impairment may contribute to but is probably not a causal element in obesity development. Nevertheless, the powerful inhibitory action on appetite and food intake of the proglucagon renders these peptides attractive in the therapy of obesity. The role of glucagon secretion in obesity development is currently uncertain, and although its actions on food intake and liver fat may be pharmacologically relevant, its further metabolic actions may be difficult to harness.

## Author contributions

The author confirms being the sole contributor of this work and has approved it for publication.

## Funding

This study was supported by NovoNordisk Foundation.

## Conflict of interest

The author declares that the research was conducted in the absence of any commercial or financial relationships that could be construed as a potential conflict of interest.

## Publisher's note

All claims expressed in this article are solely those of the authors and do not necessarily represent those of their affiliated organizations, or those of the publisher, the editors and the reviewers. Any product that may be evaluated in this article, or claim that may be made by its manufacturer, is not guaranteed or endorsed by the publisher.
